# Effects of Whole‐Body Cryotherapy Combined With Conventional Obesity Management Versus Obesity Management Alone: A Clinical Trial

**DOI:** 10.1002/oby.70019

**Published:** 2025-09-03

**Authors:** Jari E. Karppinen, Laura Suojanen, Sini Heinonen, Sanna Kaye, Birgitta W. van der Kolk, James W. White, Janne Orava, Seung Hyuk T. Lee, Eugené Dillon, Maheswary Muniandy, Aila Rissanen, Carel W. le Roux, Neil Docherty, Päivi Pajukanta, Kirsi A. Virtanen, Kirsi H. Pietiläinen

**Affiliations:** ^1^ Obesity Research Unit, Research Program for Clinical and Molecular Metabolism, Faculty of Medicine University of Helsinki Helsinki Finland; ^2^ Healthy Weight Hub, Abdominal Center Helsinki University Hospital and University of Helsinki Helsinki Finland; ^3^ Department of Internal Medicine and Rehabilitation Helsinki University Hospital Helsinki Finland; ^4^ Diabetes Complications Research Centre, School of Medicine, Conway Institute of Biomolecular and Biomedical Research University College Dublin Dublin Ireland; ^5^ Department of Biomedical Sciences, College of Medicine and Health University of Birmingham Birmingham UK; ^6^ Turku PET Centre University of Turku Turku Finland; ^7^ Turku PET Centre Turku University Hospital Turku Finland; ^8^ Department of Human Genetics, David Geffen School of Medicine at UCLA University of California Los Angeles California USA; ^9^ Mass Spectrometry Core Facility, Conway Institute of Biomolecular and Biomedical Research University College Dublin Dublin Ireland; ^10^ Bioinformatics Interdepartmental Program University of California Los Angeles California USA; ^11^ Institute for Precision Health, David Geffen School of Medicine at UCLA University of California Los Angeles California USA

**Keywords:** brown adipose tissue, cold exposure, energy metabolism, weight loss

## Abstract

**Objective:**

To investigate whether whole‐body cryotherapy (WBC) enhances weight loss, brown adipose tissue (BAT) activation, and metabolic outcomes during obesity management.

**Methods:**

Nineteen adults with obesity were assigned to a 12‐month lifestyle‐based obesity management intervention with 28 WBC sessions (−110°C, 3–4 min, ~2 × week) over the first 5 months (CRYO, *n* = 10) or the intervention without WBC (CON, *n* = 9). The primary outcome was weight loss (5 and 12 months). Secondary outcomes included BAT glucose uptake and whole‐body energy expenditure during cold stimulation (5 months), clinical parameters, subcutaneous adipose tissue transcriptomics, and skeletal muscle proteomics (5 and 12 months).

**Results:**

Weight loss in the CRYO group was 11.9% at 5 months and 9.9% at 12 months, compared to 11.5% and 8.0% in the CON group (*p* ≥ 0.54 for between‐group differences). No significant between‐group differences appeared in BAT glucose uptake, energy expenditure, adipose tissue transcriptomics, or skeletal muscle proteomics changes. However, at 5 months, the CRYO group showed greater reductions in fasting glucose (0.41 mmol/L, *p* = 0.026) and LDL cholesterol (0.44 mmol/L, *p* = 0.034).

**Conclusions:**

WBC did not significantly enhance weight loss, activate BAT, or alter most metabolic responses during conventional obesity management. Further research is needed to confirm whether WBC benefits glucose and cholesterol metabolism.

**Trial Registration:** ClinicalTrials.gov: NCT01312090

**Study Importance**

- What is already known?
  ○ Intermittent cold exposure has been suggested as a strategy for obesity treatment, partly due to its potential to activate brown adipose tissue (BAT) and increase energy expenditure.
  ○ The effects of whole‐body cryotherapy (WBC) on body weight and metabolism during conventional obesity management have not been investigated in a controlled study design.
- What does this study add?
  ○ Conventional obesity management intervention with WBC was not superior for weight loss compared with the intervention alone.
  ○ WBC, compared with obesity management alone, did not significantly increase BAT activity.
  ○ Adding WBC led to greater short‐term reductions in fasting glucose and LDL cholesterol levels.
- How might these results change the direction of research or the focus of clinical practice?
  ○ The use of WBC to enhance conventional obesity management is not supported, but other forms of cold exposure may still prove effective.
  ○ Future research should investigate whether WBC provides specific benefits for glucose and cholesterol metabolism.

## 1 Introduction

Obesity is a global health concern, contributing to the development of chronic diseases [1] and increasing early mortality [2]. Obesity management often targets a 5%–10% body weight reduction, which reduces the risk of obesity‐related comorbidities [3]. However, maintaining weight loss after dieting remains challenging, with nearly 80% of the lost weight typically regained within 5 years [4]. While hyperphagia is the main driver of weight regain, reduced energy expenditure may also contribute [5]. Therefore, identifying methods to increase energy expenditure could improve obesity management.

Cold exposure has gained interest as a tool to increase energy expenditure, partly by activating brown adipose tissue (BAT) to dissipate energy as heat [6]. However, the capacity of BAT to meaningfully increase energy expenditure is uncertain due to its small mass [7]. In contrast, skeletal muscle is the largest contributor to the cold‐induced increase in energy expenditure, even during mild cold exposure with minimal shivering [8]. Additionally, white adipose tissue (WAT) contributes to cold‐induced energy expenditure [9], suggesting that the mechanisms by which cold might influence metabolism extend beyond BAT.

Whole‐body cryotherapy (WBC), one of various forms of cold treatments, involves brief exposures (1–4 min) to extremely cold air (−110°C to −160°C). Four uncontrolled studies have explored the association between WBC and body weight [10, 11, 12, 13], with two reporting minor weight loss after 20 sessions over 4 weeks [10, 11]. However, the additive effects of WBC in a comprehensive obesity management intervention are unknown. This knowledge gap extends to other forms of cold exposure.

Mechanistically, WBC has the potential to stimulate BAT as it increases sympathetic nervous system activity [14]. However, its effects on BAT have not been studied. Similarly, the impact of WBC on tissues like WAT and skeletal muscle remains unclear as previous research has mainly used circulating biomarkers to assess its metabolic effects [15, 16]. Therefore, a more comprehensive investigation across multiple tissues is needed to better understand how WBC influences metabolism.

The primary aim of this study was to investigate whether WBC, when combined with a conventional obesity management intervention, leads to greater weight reduction than the intervention alone in adults with obesity. We hypothesized that WBC activates BAT and increases energy expenditure, thereby enhancing the efficacy of the obesity management intervention alone. Additionally, we explored whether WBC affects body composition and metabolic responses through whole‐body, blood, WAT, skeletal muscle, and liver measurements.

## 2 Methods

### 2.1 Trial Design

**FIGURE 1:**
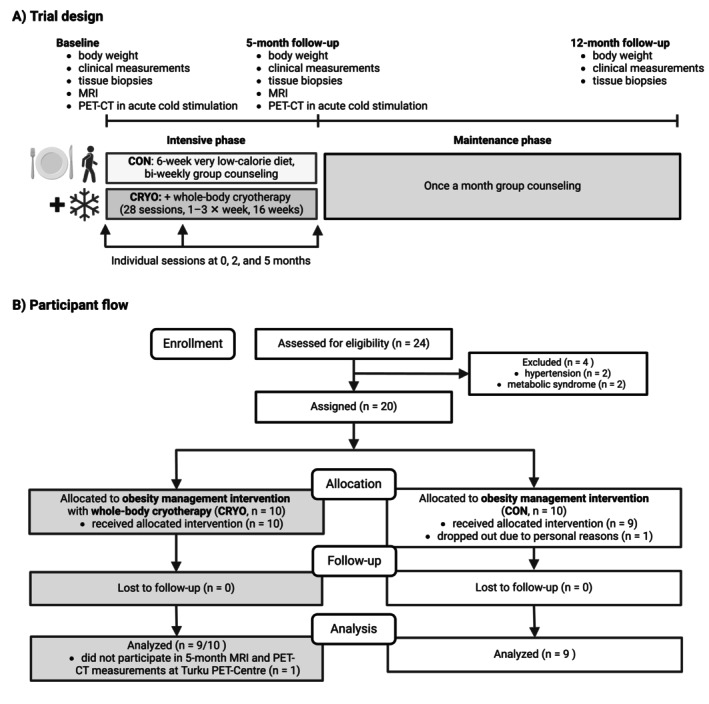
Trial profile. (A) Overall structure of the intervention and assessments of the study. (B) CONSORT flow diagram.

The Chronic Intermittent Cold Exposure on Weight Loss (CICE) study was a 12‐month parallel‐group (1:1) clinical trial (ClinicalTrials.gov identifier NCT01312090, Figure 1A), conducted at the Obesity Research Unit, University of Helsinki, Finland, and the Turku PET Centre, Turku University Hospital and University of Turku, Finland. The study received supportive statements from the ethics committees of the hospital districts of Helsinki and Uusimaa and Southwest Finland, granting ethical approval, and was conducted according to the Declaration of Helsinki. Participants provided written informed consent.

### 2.2 Participant Recruitment and Assignment

Participants were recruited through newspaper advertisement. Eligible participants were aged 18–50 years, and they had BMI > 30 kg/m^2^, body weight < 120 kg, and a history of weight loss attempts but reported stable body weight for 3 months. Inclusion criteria also included triglycerides < 3.0 mmol/L, HDL cholesterol > 0.9 mmol/L, blood pressure < 140/90 mmHg, and normal or impaired glucose tolerance. Exclusion criteria were smoking, pregnancy or breastfeeding, chronic diseases or medications affecting study outcomes, and claustrophobia or presence of ferromagnetic objects.

Participants were allocated to the treatment group (CRYO, WBC with conventional obesity management) or the active comparator group (CON, conventional obesity management only) after baseline measurements. Allocation was balanced for BMI, BAT activity, sex, and age to ensure group homogeneity, rather than using strict randomization.

### 2.3 Intervention

The intervention consisted of a 5‐month intensive phase and a 7‐month maintenance phase (Figure 1A). The intensive phase began with a 6‐week, meal‐replacement‐based very low‐calorie diet (VLCD, 500–1000 kcal/day), supplemented with low‐carbohydrate vegetables (200–250 kcal/day) and low‐fat protein sources to provide protein 70–90 g/day. Participants took daily multivitamins (Multi‐Tabs, Orion Pharma, Finland). After the VLCD phase, participants were instructed to reduce energy intake by 500–1000 kcal/day from baseline and maintain protein intake at 1.2–1.5 g/kg/day. This phase included bimonthly group sessions and three personalized individual sessions, containing behavioral therapy and diet and physical activity counseling.

Starting after group allocation, the CRYO group received 28 WBC sessions (~2 × week) over 16 weeks at Haikko Spa (Porvoo, Finland) in a three‐chamber WBC system (Univers Cryo‐Combi, Oy MJG Univers Ab, Helsinki, Finland). Supervised sessions included brief precooling in −30°C and −60°C chambers, followed by 3–4 min in a −110°C chamber. Participants wore a bathing suit, headgear, mittens, socks, and felt footwear. We monitored two early sessions, recording blood pressure, pulse, and participant feedback before and 15 min afterward. We queried adverse events at the 5‐month follow‐up.

During the maintenance phase, group sessions continued once a month. The study's dietitian (L.S.) conducted all sessions. Energy and macronutrient intakes were monitored using 3‐day food diaries and physical activity levels using the Baecke Questionnaire [17].

### 2.4 Outcomes and Assessments

Clinical measurements and tissue biopsies were performed at baseline, 5 months, and 12 months (Figure 1A). Magnetic resonance imaging (MRI) and acute cold‐stimulation measurements, including positron emission tomography combined with computed tomography (PET‐CT), were conducted at the Turku PET Centre at baseline and 5 months only to minimize radiation exposure. The 5‐month follow‐up deviated from the protocol, as measurements were conducted closer to 5 months instead of 4 months after admission due to the time required to implement the intervention. In the CRYO group, 5‐month measurements were performed within 2 weeks of the final WBC session, except for one participant who was measured 4 weeks later because of illness. Assessors were not blinded.

#### 2.4.1 Primary Outcome: Body Weight

Body weight was measured with a digital scale (seca, Hamburg, Germany) with 0.1 kg accuracy.

#### 2.4.2 Anthropometrics, Body Composition, and Circulating Biomarkers

Height was recorded at baseline to 0.1 cm using a stadiometer (seca). Waist circumference was measured to 0.5 cm with a tape measure. Fat‐free mass (FFM) and fat mass (FM) were assessed using dual‐energy X‐ray absorptiometry (Lunar Prodigy, GE Medical Systems, Madison, WI). Fasting and 2‐h oral glucose tolerance test blood samples were analyzed, and the HOMA and Matsuda indexes were calculated as previously described [18].

#### 2.4.3 Adipose Tissue Depot Masses and Liver Fat

WAT depot volumes and liver fat content were assessed using MRI and proton magnetic resonance spectroscopy (^1^H‐MRS; Gyroscan Intera CV Nova Dual, Philips Medical Systems, the Netherlands), respectively, as previously reported [19]. A T1‐weighted image at the level of the intervertebral disc L2‐L3 was used to analyze abdominal WAT volumes. Measured volumes were converted into masses using a density of 0.9196 g/mL. Liver fat content (%) represents fat relative to total liver mass, estimated from fat and water peaks in the spectrum. No validated method exists for quantifying BAT mass. We estimated BAT mass by combining optimized MRI sequences of the cervical–supraclavicular region with PET, as described previously [20]. On the fused images, we manually outlined the assumed regions of BAT, calculated volume by multiplying each traced area by the slice thickness and considering interslice gaps, and converted volume to mass using an assumed BAT density of 0.94 g/mL. However, these values should be regarded as crude estimates.

#### 2.4.4 Acute Cold Stimulation and Tissue Glucose Uptake

The measurements were performed as previously reported [21, 22]. The overnight fasted participants first stayed in a 17°C room for 2 h wearing light clothing. Cold exposure was then induced by intermittently placing one foot in 8°C water for 5 min in and out. After 40 min, glucose uptake rates for adipose tissue depots and deltoid muscle were assessed using ^18^F‐fluoro‐deoxyglucose PET‐CT (GE Discovery VCT, GE Medical Systems). Dynamic emission scanning began immediately after the 185 MBq bolus injection, sequentially imaging the clavicular, lower thoracic, and abdominal regions over 70 min. Respiratory gas exchange was measured using a Deltatrac metabolic cart (Datex‐Ohmeda, Helsinki, Finland) and a ventilated hood during the whole scanning. Average energy expenditure (kcal/day) for the period was calculated according to Péronett and Massicotte [23]. Blood pressure and heart rate were measured at the beginning and end of testing.

#### 2.4.5 WAT Biopsies, RNA Preparations, and Transcriptomics Analyses

Subcutaneous WAT biopsies were taken near the umbilicus using an open surgical technique under local lidocaine anesthesia. RNA was extracted using the AllPrep RNA/DNA/miRNA Universal Kit (QIAGEN, Nordic, Sollentuna, Sweden) with DNase I digestion. RNA quality was assessed using a 2100 Bioanalyzer (Agilent Technologies, Santa Clara, CA) before sequencing. Libraries for RNA sequencing (RNA‐seq) were prepared using the Illumina TruSeq RNA kit. Samples were sequenced on the Illumina HiSeq 2500 platform with an average sequence depth of 50–60 million paired‐end reads. RNA reads were sequenced to a length of 75 bp and aligned to the human reference genome hg38 using STAR v2.5.2b [24] and its two‐pass protocol with GENOCODE v26 annotations [25]. Sample quality was ensured using FastQC; RNA‐seq quality metrics were obtained using Picard Tools v2.20.5, and read counts were calculated with featureCounts v1.6.2 [26].

#### 2.4.6 Skeletal Muscle Biopsies, Protein Preparations, and Proteomics Analyses

Biopsies were taken from the vastus lateralis using a 5 mm Bergström needle under local lidocaine anesthesia. Approximately 15 mg of tissue was homogenized in an RIPA‐M buffer, followed by a full lyse in 8M urea. Proteins were precipitated by acetone and processed using a commercial kit (PreOmics, Germany). Peptide digestion, washing, and preparation to 0.5 g/L concentration in “LC‐LOAD” solvent were performed according to the manufacturer's instructions.

LC‐MS/MS was performed using a Q Exactive Hybrid Quadrupole‐Orbitrap Mass Spectrometer (Thermo Scientific) connected to an Ultimate 3000 RSLCnano (Dionex) ultra‐high pressure nanoflow chromatography system. Peptides were separated on an in‐house C18 column (150 nm × 0.075 mm × 3 μm, Dr. Maisch Reprosil‐Pur) over 120 min at a flow rate of 250 nL/min with a linear gradient of acetonitrile increasing from 1% to 27%. The mass spectrometer was operated in data‐dependent mode (70,000 FWHM, 300–1600 m/z), selecting the 12 most intense ions for fragmentation.

MS/MS spectra were matched against the Uniprot *Homo sapiens* database (2021_03) containing 78,120 entries using MaxQuant (version 2.0.3.0) [27]. Label‐free quantitative ion intensities were generated by specifying trypsin as the digestion enzyme while allowing two missed cleavages and a 1% false discovery rate (FDR) on peptides and proteins in searches. The data have been deposited to the ProteomeXchange Consortium via the PRIDE [28] partner repository with identifiers PXD061410 and 10.6019/PXD061410.

### 2.5 Statistical Analyses

We did not perform a priori power calculations because comparable interventions were unavailable at the time of study design. We conducted statistical analyses in R version 4.0.0 and set statistical significance at *p* < 0.05.

We analyzed the outcomes, excluding transcriptomics and proteomics, using generalized estimating equations (GEE, *geepack* package) [29]. The method is semiparametric, accounts for within‐individual correlations, and does not assume residual normality or equal variances between groups. We used group, time, and their interaction as the explanatory variables, with outcome baseline levels as covariates. We used unstructured and exchangeable correlation matrices for outcomes measured at three and two time points, respectively.

For WAT transcriptomics, we filtered transcripts with < 10 read counts across all samples and calculated normalization factors using the *edgeR* package [30]. We performed differential gene expression analysis using the *limma* package [31]. The models included participant identification to define baseline effects and treatment effects for both groups at each follow‐up. We compared changes between groups using contrasts, equivalent to group × time interactions. To adjust for technical variability, we included the first gene expression principal component as a covariate, which correlated strongly with technical factors including the percentage of uniquely mapped genes, median 5′ to 3′ prime bias, and RNA integrity number.

For skeletal muscle proteomics, we selected proteins with valid identifications, excluding those identified solely by peptides carrying modified amino acids, as well as decoy proteins marked as reverse. We log2‐transformed data and filtered out proteins not detected in at least six samples (32%) at any time point. We imputed missing values using the *minProb* method (*promor* package) [32]. For differential analysis, we used the same strategy as in WAT transcriptomics. We excluded 5‐month omics data of one CRYO group participant due to poor quality.

We performed canonical pathway analyses using QIAGEN IPA (QIAGEN Inc., https://digitalinsights.qiagen.com/IPA) [33]. For WAT, we selected transcripts with an FDR < 0.05 and used the user‐defined set as the reference. For skeletal muscle, we selected proteins with *p* < 0.05, recognizing the generally lower sensitivity of proteomics compared to transcriptomics. We used the Ingenuity Knowledge Base as the reference set, which enhances the detection of potentially activated pathways but may introduce a bias toward pathways more generally relevant than specific to the data. We considered predicted pathways with an FDR < 0.05 statistically significant and those with *z*‐scores > 2.0 (activation) or < −2.0 (inhibition) as potentially biologically meaningful.

## 3 Results

### 3.1 Participants

The trial was conducted from September 2009 to December 2010, aligning with seasonal temperature patterns to minimize variability in BAT activity. Twenty‐four individuals were assessed for eligibility, and twenty were enrolled (Figure 1B). One CON group participant dropped out before the intervention due to personal reasons. The remaining 19 participants (12 females) completed the study. One CRYO group participant withdrew from the 5‐month Turku PET‐center measurements.

Participants were aged 20–48 years and were predominantly Caucasian, with one of Caucasian‐Caribbean descent. Baseline characteristics used for allocation were comparable between groups, although the CRYO group had more variation in BMI (Table 1). MRI/PET‐estimated BAT mass was recorded in four participants per group.

**TABLE 1:** Baseline characteristics used to allocate participants to conventional obesity management intervention with whole‐body cryotherapy (CRYO) or obesity management intervention alone (CON) with *p*‐values for between‐group differences.

Characteristic	All, *n* = 19	CRYO, *n* = 10	CON, *n* = 9	*p*
Sex				1.0
Male	7 (37%)	4 (40%)	3 (33%)	
Female	12 (63%)	6 (60%)	6 (67%)	
Age, years	35.2 (7.8)	36.3 (7.5)	34.0 (8.4)	0.54
Body weight, kg	99.0 (14.0)	100.4 (9.8)	97.4 (18.1)	0.66
Height, cm	168.7 (9.7)	170.1 (8.5)	167.1 (11.1)	0.51
BMI, kg/m^2^	34.6 (2.7)	34.7 (2.4)	34.6 (3.1)	0.94
BAT glucose uptake, μmol × kg × min	4.95 (6.37)	4.70 (5.84)	5.71 (7.34)	0.49

*Note*: Data given as *n* (%) or mean (SD). Between‐group comparisons were analyzed using linear or logistic regression. BAT glucose uptake was ln‐transformed for the analysis.

Abbreviation: BAT, brown adipose tissue.

### 3.2 Participants Adhered Well to the Intervention

Both groups reduced energy intake from fats and carbohydrates by ~750 kcal/day from baseline to 5 months (Table 2). By 12 months, energy intake had returned toward baseline. Both groups increased physical activity during the intensive phase, and the increase largely persisted to 12 months.

**TABLE 2:** Self‐reported dietary intake based on 3‐day food diaries and physical activity based on Baecke questionnaire scores in participants allocated to conventional obesity management intervention with whole‐body cryotherapy (CRYO) or obesity management intervention alone (CON).

	CRYO, *n* = 10			CON, *n* = 9		
	Baseline	5 months	12 months	Baseline	5 months	12 months
Dietary intake						
Energy, kcal/day	2369 (1146)	1637 (554)**	1842 (451)	2246 (694)	1462 (281)***	2143 (854)
Fat, g	89 (45)	45 (16)***	62 (18)*	85 (32)	46 (23)***	77 (41)
Carbohydrate, g	262 (140)	171 (62)**	193 (53)	263 (73)	159 (33)***	228 (88)
Protein, g	99 (46)	97 (35)	107 (40)	85 (25)	88 (35)	105 (27)**
Fat, %	33 (5)	24 (5)**	30 (6)	33 (4)	28 (11)	30 (6)
Carbohydrate, %	45 (5)	43 (8)	42 (5)	48 (6)	45 (7)	43 (7)
Protein, %	17 (2)	24 (4)***	23 (6)**	16 (3)	24 (7)***	21 (7)*
Baecke physical activity index						
Total, 0–15	7.9 (1.6)	9.1 (1.7)***	9.0 (1.4)**	8.0 (1.1)	8.9 (0.8)***	8.7 (1.2)
Work, 0–5	2.3 (0.7)	2.3 (0.7)	2.3 (0.6)	3.0 (0.5)	3.2 (0.7)	3.3 (0.4)*
Sport, 0–5	2.6 (0.5)	3.1 (0.9)*	3.2 (0.6)***	2.3 (0.5)	2.8 (0.5)*	2.4 (0.6)
Leisure‐time, 0–5	2.9 (0.7)	3.5 (0.5)***	3.2 (0.7)	2.8 (0.5)	3.1 (0.7)	3.2 (0.8)**

*Note*: There were no statistically significant between‐group differences in changes in any variable. Data reported as mean (SD). One participant in both groups had missing dietary records at 12 months. Baecke physical activity total and sport index scores were missing for one participant in both groups at baseline and for one CRYO group participant at 12 months. Statistical tests were performed using generalized estimating equations models using unstructured correlation matrix. ***p < 0.05, **p < 0.01, *p < 0.05, for within‐group changes from baseline.

CRYO participants attended a median of 25.5 out of 28 WBC sessions; one attended 21 and three completed all. Missed sessions were due to acute respiratory infections and travel. Two CRYO participants received antibiotics for acute respiratory infections during the intensive phase, and one began thyroxine treatment at 7 months. No serious adverse events occurred. Mild events included evening coldness (*n* = 2), headaches after early sessions (*n* = 1), and nasal numbness lasting ≤ 24 h (*n* = 1). Fifteen minutes after the monitored WBC session, systolic blood pressure and pulse rate were lower, and participants reported less tiredness and an improved mood compared to before the session (Table S5).

### 3.3 Body Weight Loss Did Not Significantly Differ Between Groups

**FIGURE 2:**
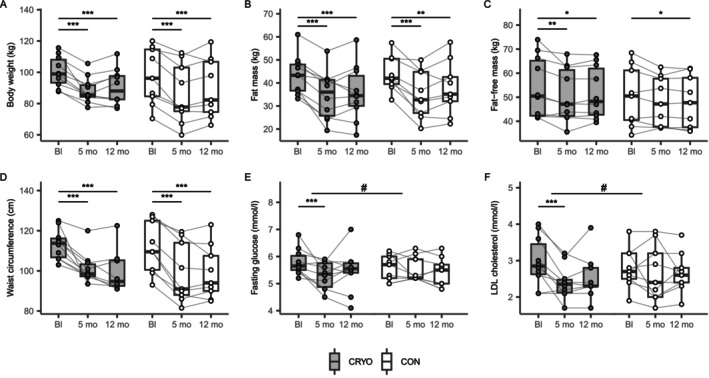
Whole‐body cryotherapy with conventional obesity management intervention (CRYO, *n* = 10) did not significantly enhance body weight loss compared to obesity management intervention alone (CON, *n* = 9). The intervention effects on (A) primary outcome, body weight, (B) fat mass, (C) fat‐free mass, (D) waist circumference, (E) fasting glucose, and (F) LDL cholesterol. The results were analyzed using generalized estimating equation models, adjusted for outcome baseline (Bl) values. *p < 0.05, **p < 0.01, ***p < 0.001, for within‐group change; ^#^ *p* < 0.05, for between‐group difference in change from baseline.

Both groups successfully lost body weight, with no significant differences (Table 3, Figure 2A). Weight loss in the CRYO group was 11.9 kg (11.9%) at 5 months and 9.9 kg (9.9%) at 12 months, compared to 11.2 kg (11.5%) at 5 months and 7.8 kg (8.0%) at 12 months in the CON group.

**TABLE 3:** Weight loss and changes in body composition and clinical metabolic outcomes in response to conventional obesity management intervention with whole‐body cryotherapy (CRYO) compared with obesity management intervention alone (CON).

	CRYO, *n* = 10			CON, *n* = 9			CRYO vs. CON	
Variable	Mean (SD)	Change (95% CI)	*p*	Mean (SD)	Change (95% CI)	*p*	Difference in change (95% CI)	*p*
Primary outcome								
Body weight, kg								
Baseline	100.4 (9.8)			97.4 (18.1)				
5 months	88.5 (8.4)	−11.9 (−15.6 to −8.2)	< 0.001	86.2 (19.2)	−11.2 (−14.3 to −8.1)	< 0.001	−0.7 (−5.6 to 4.1)	0.77
12 months	90.5 (11.5)	−9.9 (−15.0 to −4.8)	< 0.001	89.6 (18.9)	−7.8 (−12.3 to −3.3)	< 0.001	−2.1 (−8.9 to 4.6)	0.54
Secondary outcomes								
Body composition and anthropometrics								
Fat mass, kg								
Baseline	43.4 (8.5)			44.0 (7.6)				
5 months	34.7 (10.5)	−8.7 (−11.2 to −6.2)	< 0.001	34.9 (10.1)	−9.1 (−11.9 to −6.3)	< 0.001	0.4 (−3.3 to 4.2)	0.82
12 months	35.7 (12.0)	−7.7 (−11.1 to −4.2)	< 0.001	37.7 (11.6)	−6.3 (−10.5 to −2.1)	0.003	−1.3 (−6.8 to 4.1)	0.63
Fat‐free mass, kg								
Baseline	54.1 (12.7)			50.0 (12.5)				
5 months	50.8 (11.1)	−3.3 (−5.4 to −1.1)	0.003	48.2 (10.2)	−1.8 (−3.8 to 0.2)	0.081	−1.5 (−4.4 to 1.5)	0.32
12 months	51.7 (10.9)	−2.4 (−4.3 to −0.4)	0.016	48.0 (10.9)	−2.0 (−3.8 to −0.2)	0.030	−0.4 (−3.1 to 2.2)	0.76
Body fat percentage, %								
Baseline	43.6 (9.0)			45.8 (4.1)				
5 months	39.2 (11.2)	−4.4 (−6.5 to −2.4)	< 0.001	40.1 (5.3)	−5.7 (−8.4 to −2.9)	< 0.001	1.2 (−2.2 to 4.7)	0.48
12 months	39.3 (11.1)	−4.3 (−6.5 to −2.1)	< 0.001	42.1 (7.1)	−3.7 (−6.8 to −0.5)	< 0.001	−0.6 (−4.5 to 3.2)	0.75
Waist circumference, cm								
Baseline	113.2 (7.4)			111.8 (12.9)				
5 months	101.3 (7.4)	−12.0 (−16.3 to −7.6)	< 0.001	98.8 (14.8)	−12.9 (−16.0 to −9.9)	< 0.001	1.0 (−4.3 to 6.3)	0.71
12 months	99.9 (10.8)	−14.2 (−18.9 to −9.5)	< 0.001	99.0 (13.2)	−12.8 (−16.9 to −8.6)	< 0.001	−1.4 (−7.7 to 4.9)	0.66
Glucose metabolism								
Fasting glucose, mmol/L								
Baseline	5.79 (0.48)			5.59 (0.47)				
5 months	5.30 (0.49)	−0.49 (−0.76 to −0.22)	< 0.001	5.51 (0.45)	−0.08 (−0.32 to 0.17)	0.54	−0.41 (−0.77 to −0.05)	0.026
12 months	5.47 (0.78)	−0.32 (−0.70 to 0.06)	0.10	5.41 (0.54)	−0.18 (−0.38 to 0.02)	0.080	−0.14 (−0.57 to 0.29)	0.52
Fasting insulin, IU/L								
Baseline	8.73 (4.13)			10.61 (5.13)				
5 months	8.34 (5.18)	−0.39 (−4.25 to 3.47)	0.84	7.88 (4.38)	−2.73 (−3.66 to −1.80)	< 0.001	2.34 (−1.63 to 6.31)	0.25
12 months	6.68 (3.24)	−2.05 (−4.38 to 0.28)	0.085	10.90 (6.34)	0.29 (−1.79 to 2.36)	0.79	−2.34 (−5.46 to 0.78)	0.14
HOMA index								
Baseline	2.28 (1.22)			2.70 (1.48)				
5 months	2.00 (1.37)	−0.28 (−1.36 to 0.80)	0.61	1.97 (1.17)	−0.74 (−1.09 to −0.38)	< 0.001	0.46 (−0.68 to 1.60)	0.43
12 months	1.68 (0.89)	−0.61 (−1.34 to 0.13)	0.11	2.71 (1.77)	0.01 (−0.58 to 0.60)	0.97	−0.62 (−1.56 to 0.33)	0.20
2‐h glucose, mmol/L								
Baseline	6.78 (1.82)			6.23 (1.40)				
5 months	5.33 (0.68)	−1.45 (−2.32 to −0.58)	0.001	5.54 (1.06)	−0.69 (−1.77 to 0.38)	0.21	−0.76 (−2.15 to 0.62)	0.28
12 months	5.41 (1.41)	−1.37 (−2.08 to −0.66)	0.002	5.70 (1.02)	−0.53 (−1.41 to 0.34)	0.23	−0.84 (−1.96 to 0.29)	0.15
Matsuda index								
Baseline	5.96 (4.05)			4.62 (2.67)				
5 months	7.85 (4.31)	1.89 (−0.26 to 4.04)	0.085	7.73 (4.54)	3.11 (1.22 to 5.00)	0.001	−1.22 (−4.08 to 1.64)	0.40
12 months	9.63 (7.00)	3.41 (−0.79 to 7.61)	0.11	5.55 (3.79)	0.77 (−0.06 to 1.60)	0.067	2.63 (−1.65 to 6.92)	0.23
Serum lipids								
Total cholesterol, mmol/L								
Baseline	4.67 (0.75)			4.56 (0.67)				
5 months	4.04 (0.59)	−0.63 (−0.93 to −0.33)	< 0.001	4.33 (0.83)	−0.22 (−0.58 to 0.14)	0.23	−0.41 (−0.88 to 0.06)	0.088
12 months	4.34 (0.69)	−0.33 (−0.62 to −0.04)	0.024	4.49 (0.71)	−0.07 (−0.46 to 0.32)	0.74	−0.26 (−0.75 to 0.22)	0.29
LDL cholesterol, mmol/L								
Baseline	2.97 (0.67)			2.79 (0.55)				
5 months	2.40 (0.45)	−0.57 (−0.81 to −0.33)	< 0.001	2.66 (0.77)	−0.13 (−0.45 to 0.19)	0.41	−0.44 (−0.84 to −0.03)	0.034
12 months	2.52 (0.63)	−0.46 (−0.72 to −0.20)	< 0.001	2.66 (0.55)	−0.13 (−0.40 to 0.13)	0.33	−0.33 (−0.70 to 0.05)	0.086
HDL cholesterol, mmol/L								
Baseline	1.35 (0.33)			1.40 (0.29)				
5 months	1.40 (0.26)	0.06 (−0.07 to 0.19)	0.38	1.42 (0.25)	0.03 (−0.10 to 0.15)	0.68	0.03 (−0.15 to 0.21)	0.73
12 months	1.45 (0.27)	0.11 (−0.01 to 0.23)	0.078	1.58 (0.30)	0.13 (−0.01 to 0.27)	0.068	−0.02 (−0.21 to 0.16)	0.80
Triglycerides, mmol/L								
Baseline	1.09 (0.35)			1.26 (0.61)				
5 months	0.76 (0.40)	−0.33 (−0.56 to −0.09)	0.006	0.80 (0.25)	−0.46 (−0.77 to −0.16)	0.003	0.14 (−0.25 to 0.52)	0.49
12 months	0.82 (0.34)	−0.27 (−0.50 to 0.04)	0.020	0.97 (0.40)	−0.29 (−0.61 to 0.04)	0.082	0.02 (−0.38 to 0.41)	0.93
Inflammation								
Leukocytes, E9/L								
Baseline	5.93 (0.19)			5.72 (1.18)				
5 months	5.81 (1.53)	−0.12 (−0.46 to 0.22)	0.49	5.79 (0.77)	0.07 (−0.55 to 0.69)	0.83	−0.19 (−0.89 to 0.52)	0.60
12 months	5.44 (0.92)	−0.49 (−1.01 to 0.03)	0.062	5.59 (1.07)	−0.13 (−0.89 to 0.62)	0.73	−0.36 (−1.27 to 0.56)	0.45

*Note*: The results are from generalized estimating equation models with unstructured correlation matrix and adjusted for outcome baseline levels. CRYO versus CON (group × time interaction) presents the treatment effect of added whole‐body cryotherapy.

### 3.4 Body Composition and Circulating Metabolic Markers Improved in Both Groups

Changes in FFM, FM, and waist circumference were not significantly different between groups (Figure 2B–D). Circulating metabolic markers improved in both groups during the intensive phase, although most changes partially reversed by 12 months (Table 3). The CRYO group showed a 0.41 mmol/L greater decrease in fasting glucose and a 0.44 mmol/L greater decrease in LDL cholesterol at 5 months than the CON group (Figure 2E,F), but differences were no longer significant at 12 months.

### 3.5 WBC Did Not Significantly Activate BAT but Preserved Deltoid Glucose Uptake

**FIGURE 3:**
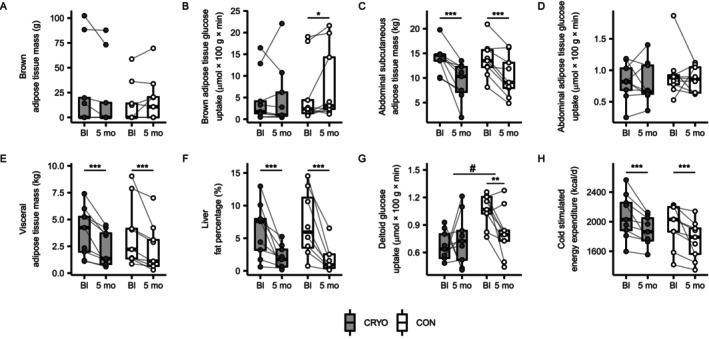
Whole‐body cryotherapy with conventional obesity management intervention (CRYO, *n* = 9) did not significantly activate brown adipose tissue (BAT) during acute cold stimulation compared to obesity management intervention alone (CON, *n* = 9). The intervention effects on (A) BAT mass and (B) glucose uptake, (C) subcutaneous white adipose tissue mass and (D) glucose uptake, (E) visceral adipose tissue mass, (F) liver fat percentage, (G) deltoid glucose uptake, and (H) energy expenditure. The results were analyzed using generalized estimating equation models, adjusted for outcome baseline (Bl) values. *p < 0.05, **p < 0.01, ***p < 0.001, for within‐group change; ^#^ *p* < 0.05, for between‐group difference in change from baseline.

We found no robust evidence that WBC activates BAT (Table 4). At 5 months, MRI‐PET estimated BAT mass was recorded in three CRYO participants and five CON participants (Figure 3A). BAT glucose uptake significantly increased in the CON group, with no significant between‐group difference (Figure 3B). Energy expenditure decreased from baseline to 5 months in both groups by ~190 kcal/day (Figure 3H). FFM‐ and FM‐adjusted decreases were 88 kcal/day (*p* = 0.084) in the CRYO group and 85 kcal/day (*p* = 0.32) in the CON group.

**TABLE 4:** Changes in adipose tissue depot masses, liver fat, and physiological outcomes during acute cold stimulation in response to conventional obesity management with whole‐body cryotherapy (CRYO) compared with obesity management alone (CON).

	CRYO, *n* = 9			CON, *n* = 9			CRYO vs. CON	
Variable	Mean (SD)	Change (95% CI)	*p*	Mean (SD)	Change (95% CI)	*p*	Difference in change (95% CI)	*p*
Adipose tissue depot masses, kg								
Whole‐body subcutaneous								
Baseline	40.5 (8.5)			40.4 (6.9)				
5 months	33.5 (10.1)	−7.0 (−9.3 to −4.6)	< 0.001	32.3 (8.6)	−8.1 (−11.0 to −5.2)	< 0.001	1.1 (−2.6 to 4.8)	0.56
Abdominal subcutaneous								
Baseline	14.0 (2.9)			13.7 (3.6)				
5 months	9.2 (3.7)	−4.8 (−6.8 to −2.7)	< 0.001	10.4 (4.2)	−3.3 (−4.6 to −2.1)	< 0.001	−1.5 (−3.9 to 0.9)	0.23
Visceral								
Baseline	3.9 (2.3)			3.6 (3.0)				
5 months	2.3 (1.6)	−1.6 (−2.2 to −0.9)	< 0.001	2.3 (2.2)	−1.3 (−2.1 to −0.4)	0.004	−0.3 (−1.4 to 0.8)	0.57
Liver fat, %								
Baseline	6.2 (4.1)			7.2 (5.0)				
5 months	2.0 (1.8)	−4.2 (−6.4 to −2.0)	< 0.001	1.6 (2.1)	−5.6 (−8.0 to −3.2)	< 0.001	1.4 (−1.9 to 4.6)	0.42
Glucose uptake rates, μmol × kg × min								
Brown adipose tissue								
Baseline	4.70 (5.84)			5.71 (7.34)				
5 months	5.07 (7.25)	0.37 (−2.03 to 2.78)	0.76	7.85 (8.31)	2.14 (0.13 to 4.15)	0.037	−1.77 (−4.91 to 1.37)	0.27
Abdominal subcutaneous adipose tissue								
Baseline	0.82 (0.29)			0.93 (0.38)				
5 months	0.80 (0.33)	−0.02 (−0.19 to 0.14)	0.78	0.86 (0.20)	−0.07 (−0.29 to 0.14)	0.50	0.05 (−0.22 to 0.32)	0.72
Visceral adipose tissue								
Baseline	2.04 (0.69)			2.28 (0.52)				
5 months	1.80 (0.44)	−0.24 (−0.60 to 0.12)	0.19	2.36 (0.40)	0.08 (−0.19 to 0.36)	0.55	−0.32 (−0.77 to 0.13)	0.16
Cervical adipose tissue								
Baseline	0.70 (0.28)			1.09 (0.61)				
5 months	0.79 (0.24)	0.09 (−0.09 to 0.28)	0.31	0.83 (0.25)	−0.25 (−0.58 to 0.08)	0.13	0.35 (−0.03 to 0.72)	0.071
Perirenal adipose tissue								
Baseline	2.00 (0.50)			2.39 (0.93)				
5 months	1.90 (0.33)	−0.10 (−0.45 to 0.24)	0.55	1.93 (0.48)	−0.46 (−1.14 to 0.21)	0.18	0.36 (−0.40 to 1.12)	0.36
Deltoid muscle								
Baseline	0.67 (0.16)			1.06 (0.17)				
5 months	0.74 (0.28)	0.07 (−0.17 to 0.31)	0.56	0.81 (0.27)	−0.25 (−0.41 to −0.09)	0.002	0.32 (0.03 to 0.62)	0.029
Physiological measures before cold stimulation								
Systolic blood pressure, mmHg								
Baseline	127 (9)			121 (15)				
5 months	127 (13)	0 (−6 to 6)	0.95	116 (11)	−6 (−10 to −1)	0.012	5 (−2 to 13)	0.16
Diastolic blood pressure, mmHg								
Baseline	82 (6)			80 (16)				
5 months	80 (8)	−2 (−6 to 1)	0.24	78 (9)	−3 (−10 to 4)	0.39	1 (−7 to 8)	0.85
Heart rate, bpm								
Baseline	65 (9)			64 (7)				
5 months	61 (9)	−5 (−9 to 0)	0.033	60 (6)	−4 (−9 to 1)	0.15	−1 (−8 to 5)	0.76
Physiological measures during or at the end of cold stimulation								
Systolic blood pressure, mmHg								
Baseline	142 (19)			128 (16)				
5 months	133 (18)	−9 (−15 to −2)	0.013	123 (12)	−5 (−10 to 0)	0.042	−4 (−12 to 5)	0.41
Diastolic blood pressure, mmHg								
Baseline	91 (8)			84 (13)				
5 months	82 (10)	−9 (−14 to −3)	0.001	81 (7)	−2 (−7 to 1)	0.18	−6 (−13 to 1)	0.091
Heart rate, bpm								
Baseline	64 (9)			62 (6)				
5 months	57 (9)	−7 (−10 to −4)	0.002	58 (8)	−4 (−7 to 0)	0.053	−4 (−8 to 1)	0.12
Energy expenditure, kcal/day								
Baseline	2076 (302)			1938 (285)				
5 months	1878 (195)	−198 (−296 to −100)	< 0.001	1750 (259)	−188 (−301 to −74)	0.001	−11 (−161 to 140)	0.89
Respiratory exchange ratio								
Baseline	0.77 (0.02)			0.78 (0.02)				
5 months	0.77 (0.02)	0.00 (−0.02 to 0.02)	0.93	0.80 (0.02)	0.02 (0.01 to 0.02)	< 0.001	−0.02 (−0.03 to 0.00)	0.061

*Note*: The results are from generalized estimating equation models with exchangeable correlation matrix and adjusted for outcome baseline levels. CRYO versus CON (group × time interaction) presents the treatment effect of added whole‐body cryotherapy.

WAT depot masses and liver fat decreased in both groups from baseline to 5 months (Figure 3C,E,F). Glucose uptake did not significantly change in abdominal subcutaneous WAT (Figure 3D) or other WAT depots (Table 4). However, deltoid muscle glucose uptake changed differently between groups, mainly due to a decrease in the CON group (Figure 3G).

### 3.6 WAT Transcriptomics Revealed Downregulation of Mitochondrial Pathways in Both Groups

Both groups showed largely comparable WAT gene expression trajectories, with no significant between‐group differences across 19,287 analyzed transcripts at 5 and 12 months (FDR < 0.05, Table S1).

**FIGURE 4:**
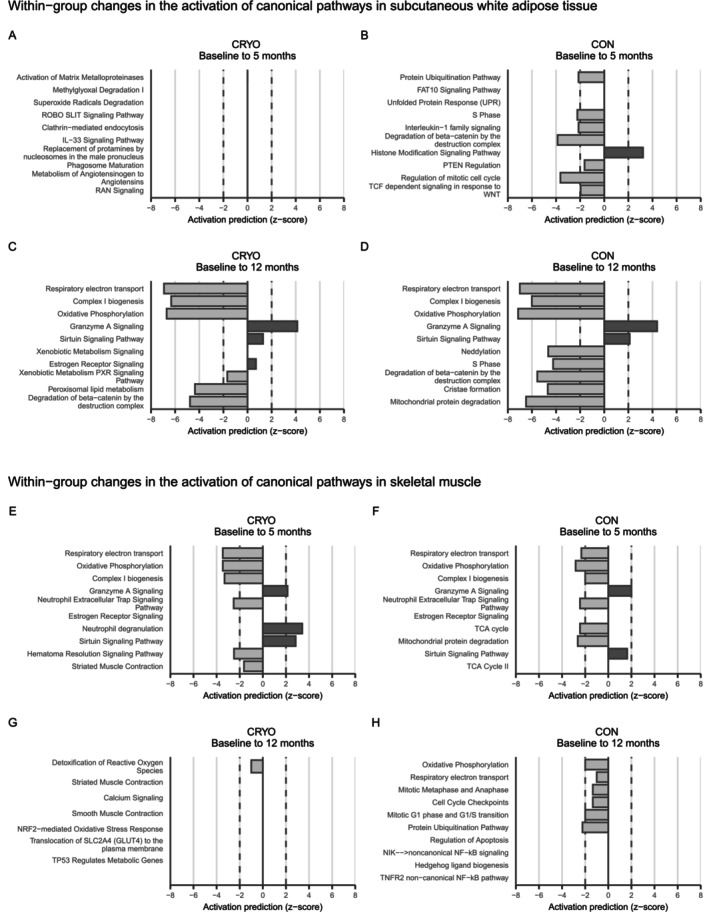
Downregulation of particularly mitochondria‐related biological pathways in abdominal subcutaneous white adipose tissue and vastus lateralis muscle occurred both in participants receiving conventional obesity management intervention with whole‐body cryotherapy (CRYO, *n* = 10) and obesity management intervention alone (CON, *n* = 9). (A, B) The top 10 enriched biological pathways in subcutaneous adipose tissue within groups based on differentially expressed genes from baseline to 5 months. (C, D) The top 10 enriched biological pathways in subcutaneous adipose tissue within groups based on differentially expressed genes from baseline to 12 months. (E, F) Explorative top 10 enriched biological pathways in skeletal muscle within groups based on nominally differentially expressed proteins (*p* < 0.05) from baseline to 5 months. (G, H) Explorative top 10 enriched biological pathways in skeletal muscle within groups based on nominally differentially expressed proteins (*p* < 0.05) from baseline to 12 months. For several pathways, IPA provided *z*‐scores for pathway directionality by calculating the observed number of “activated” genes (*z*‐score > 0), “inhibited” genes (*z*‐score < 0), or no directionality prediction (*z*‐score = 0). *Z*‐scores > 2 or < −2 suggest potentially biologically meaningful activation or inhibition.

At 5 months, 29 transcripts were differentially expressed in the CRYO group and 1192 in the CON group (Table S1). Pathway analysis showed potential meaningful activation or inhibition only in the CON group, suggesting reduced cellular growth, protein synthesis, and inflammation (Figure 4A,B).

At 12 months, 4641 transcripts were differentially expressed in the CRYO group and 5065 in the CON group. Downregulation of mitochondrial metabolism emerged as the strongest finding in both groups (Figure 4C,D). Full pathway analysis results are shown in Table S2.

### 3.7 Skeletal Muscle Proteomics Revealed Mitochondrial Pathway Inhibition in Both Groups

Of the 1013 analyzed proteins, 123 were differently expressed (*p* < 0.05) in the CRYO group and 64 in the CON group at 5 months, with 123 proteins showing different trajectories between groups (Table S3). Inhibition of mitochondrial pathways appeared as the most potentially altered biological process in both groups (Figure 4C,D). Between‐group comparisons showed only the *Neutrophil Degranulation* pathway to be more meaningfully upregulated (*z*‐score 2.0) in the CRYO group compared with the CON group (Table S4).

At 12 months, 57 proteins in the CRYO group and 33 in the CON group showed nominally significant changes from baseline, while 43 proteins showed different trajectories between groups (Table S3). Downregulation of mitochondrial metabolism pathways was weakly observed in the CON group (Figure 4G,H). Between‐group comparisons predicted no pathway activation or inhibition considered potentially biologically meaningful (Table S4).

## 4 Discussion

This study found that a conventional obesity management intervention with 28 WBC sessions was not superior to the intervention alone for weight loss in adults with obesity. WBC did not significantly increase BAT activity during cold stimulation. Metabolic responses to dieting followed largely similar trajectories between groups, with mitochondrial pathway downregulation emerging as a key feature in both WAT and skeletal muscle. Despite the lack of robust long‐term effects, WBC showed potential short‐term benefits by reducing fasting glucose and LDL cholesterol while preserving deltoid glucose uptake during dieting. Therefore, WBC may exert some metabolic effects that warrant further research.

This trial is, to our knowledge, the first controlled study in humans to investigate WBC—or any form of cold exposure—as an adjunct to an obesity management intervention. While two previous uncontrolled studies reported minor body weight loss during WBC treatment [10, 11], we found no statistical evidence that WBC enhances weight loss. Previous monthlong cold exposure studies, in which participants ate freely and either slept in 19°C room temperature [34] or underwent five 2‐h sessions per week wearing a cooling suit circulating 10°C water [35], showed stable body weight. Therefore, current evidence does not support cold exposure as a standalone or adjunct weight loss tool.

While BAT activity appeared to increase across the whole sample during weight loss, we found no observable evidence that WBC provided benefits. This contradicts our hypothesis and may explain why WBC did not notably enhance weight loss. However, this result should not be generalized to all forms of cold exposure. Previous studies increased BAT activity with daily 2‐ to 10‐h cold exposure sessions over 10 days [36, 37, 38] to 1 month [34, 35], contrasting with short exposures in this study. Similarly, WBC did not significantly alter energy expenditure during acute cold stimulation. Because the measure reflects both resting energy expenditure and the cold‐induced increment, and we did not perform measurements at thermoneutrality, we could not separate their contributions.

Along with global sympathetic nervous system activation [14], WBC may alter WAT and skeletal muscle temperatures, potentially influencing molecular processes in these tissues. For example, decreases in skin, muscle, and rectal temperatures were not significantly different between a 4‐min WBC session and a 4‐min immersion in 8°C water [39]. However, we found no robust evidence that WBC modulated weight loss‐associated molecular changes in WAT or skeletal muscle. Previous research has linked dieting to mostly downregulated mitochondrial metabolism in the WAT transcriptome [40]. Our results suggest that WBC did not counteract this downregulation, despite cold exposure upregulating mitochondrial gene expression in mice [41]. While dieting does not typically downregulate mitochondrial metabolism in skeletal muscle [40], our exploratory findings suggest that downregulation at the proteome level after the intensive phase was not mitigated by WBC.

WBC showed some short‐term benefits by improving fasting glucose and LDL cholesterol levels compared to weight loss alone during the intensive phase. However, these benefits did not last until the end of the study. The greater improvement in fasting glucose is most aligned with previous evidence, as a greater decline was observed in healthy men without obesity after nine WBC sessions compared with control participants [42]. Although improvements in glucose metabolism after cold exposure are often attributed to increased BAT activation, skeletal muscle adaptations are a more likely explanation [43]. We observed a decline in deltoid muscle glucose uptake in the CON group compared with the CRYO group, possibly because WBC prevented dieting‐induced adaptations. However, since the deltoid is a nonshivering muscle with minimal increases in glucose uptake [21] or oxygen consumption [8] during cold stimulation, this finding may not be relevant.

The LDL cholesterol improvements are partly consistent with previous studies [15]. In mice, cold‐induced BAT activation lowers circulating cholesterol by enhancing triglyceride uptake from VLDL particles, promoting faster remnant formation and clearance by the liver [44]. However, a previous 1‐month cold exposure trial that increased BAT activation did not observe significant reductions in LDL cholesterol levels [34].

This study demonstrates the feasibility of WBC as an adjunct to a lifestyle‐based weight loss program, as participants adhered well to the treatment. Our dosing regimen (~3–8 min per week for 16 weeks) mirrors typical real‐world practice, giving the findings strong ecological validity. The main limitation, however, is that such dosing may be insufficient to elicit observable physiological adaptations, and we cannot exclude the possibility that more frequent administration and/or a longer‐lasting intervention would prove effective. A second limitation is the small sample size, which may have restricted our ability to detect anything other than large between‐group differences, resulting in uncertainty in our conclusions. The sample size also limited the analysis of moderating effects of age and sex; however, the groups were balanced for these characteristics. The third important limitation concerns the acute cold‐stimulation method: immersing one foot in cold water is no longer recommended because noxious cold‐receptor stimulation may evoke central inhibitory effects on BAT thermogenesis [45]. We also cannot exclude the possibility that cold acclimation in the CRYO group masked WBC effects, as we did not measure body temperatures or muscle activity during acute cold stimulation. Nevertheless, changes in energy expenditure, blood pressure, and heart rate appeared largely similar between groups. Additional limitations are that we did not standardize the menstrual cycle phase, and BAT assessment was limited to glucose uptake. We also did not adjust the secondary outcomes for multiple comparisons; therefore, the observed benefits of WBC should be considered exploratory.

## 5 Conclusion

WBC combined with a conventional obesity management intervention did not produce significantly greater body weight loss compared with the intervention alone in adults with obesity. Secondary outcomes showed largely similar body composition and metabolic responses between the two groups. Specifically, the hypothesized role of WBC in activating BAT was not supported. While WBC may lower fasting glucose and LDL cholesterol, these results need further confirmation. These findings apply only to WBC and should not be generalized to other cold exposure methods.

## Conflicts of Interest

S.H. declares advisory board membership for Novo Nordisk. S.K. declares support for attending meetings and/or travel from Novo Nordisk, Sanofi, Nordic Infucare, and Medtronic; and stock ownership of Sanofi until 2024. C.W.L.R. declares grants from the Irish Research Council, Health Research Board, and Science Foundation Ireland; consulting fees/presentation fees/support for attending meetings and/or travel from Novo Nordisk, Eli Lilly, Johnson & Johnson, Boehringer Ingelheim, GI Dynamics, Herbalife, Altimmune, Irish Life Health, Amgen, Arrowhead, Roche, AstraZeneca, Keyron, Gila Pharmaceuticals, Rhythm Pharmaceuticals, and Currax Pharmaceuticals; an unpaid role in the Irish Society for Nutrition and Metabolism; and co‐ownership of My Best Weight and Beyond BMI obesity clinics. K.H.P. declares advisory board membership for Boehringer Ingelheim, Eli Lilly, Novo Nordisk, and Vivus and presentation fees from AstraZeneca, Eli Lilly, GlaxoSmithKline, Novo Nordisk, Orion, and UCB. The other authors declare no conflicts of interest.

## Supporting information


**Data S1:** Tables.

**Table S5:** Cardiovascular vital signs and subjective experiences before and ~15 min after whole‐body cryotherapy (WBC) treatment. The data were collected during first (*n* = 3) or third (*n* = 5) organized WBC session. Two participants had not started the intervention; therefore, the data are from eight participants. The results are medians with interquartile ranges and inferential testing was performed using Wilcoxon Signed Rank Test.

## Data Availability

The data that support the findings of this study are available on request from the corresponding author. The data are not publicly available due to privacy or ethical restrictions.
