# Supplementary material for: Effects of Whole‐Body Cryotherapy Combined With Conventional Obesity Management Versus Obesity Management Alone: A Clinical Trial
Source: Obesity (Silver Spring). 2025 Sep 3;33(11):2112–27. doi: 10.1002/oby.70019 (PMC12559775; doi:10.1002/oby.70019)
Supplement: Supplementary file 2 — Table S5: Cardiovascular vital signs and subjective experiences before and ~15 min after whole‐body cryotherapy (WBC) treatment. The data were collected during first (n = 3) or third (n = 5) organized WBC session. Two participants had not started the intervention; therefore, the data are from eight participants. The results are medians with interquartile ranges and inferential testing was performed using Wilcoxon Signed Rank Test. [file OBY-33-2112-s001.docx]

**Table S5** Cardiovascular vital signs and subjective experiences before and ~15 min after whole-body cryotherapy (WBC) treatment. The data were collected during first (n=3) or third (n=5) organized WBC session. Two participants had not started the intervention; therefore, the data are from eight participants. The results are medians with interquartile ranges and inferential testing was performed using Wilcoxon Signed Rank Test.

| **Variable** | **Before WBC** | **After WBC** | **Change** | **P-value** |
| --- | --- | --- | --- | --- |
| ***Cardiovascular vital signs (n=8)*** |  |  |  |  |
| Systolic blood pressure, mmHg | 145 (131–156) | 127 (122–143) | -15 (-17– -11) | 0.030 |
| Diastolic blood pressure, mmHg | 92 (90–99) | 92 (88–94) | -3 (-12–2) | 0.44 |
| Pulse, beats per minutes | 82 (78–87) | 71 (61–77) | -9 (-14–3) | 0.039 |
| ***Visual analog scale 0–100 (n=7)*** |  |  |  |  |
| 1. How warm did you feel? (0 = very warm, 100 = ice cold) | 35 (21–42) | 19 (8–47) | -1 (-15–15) | 0.87 |
| 1. How hungry were you? (0 = satieted, 100 = very hungry) | 9 (4–50) | 11 (4–34) | 0 (-3–2) | 0.79 |
| 1. How tired did you feel? (0 = alert, 100 = very tired) | 65 (27–69) | 17 (11–24) | -21 (-57– -1) | 0.03 |
| 1. How painful did your back feel? (0 = painless, 100 = very painful) | 0 (0–2) | 0 (0–2) | 0 (0– 0) | 1 |
| 1. How painful did your joints feel? (0 = painless, 100 = very painful) | 0 (0–8) | 1 (0–7) | 0 (0–0) | 1 |
| 1. How dry did your skin feel? (0 = normal, 100 = dry) | 7 (2–9) | 7 (1–9) | 0 (-10–0) | 0.20 |
| 1. How good did you feel? (0 = good, 100 = bad) | 22 (15–36) | 5 (3–16) | -18 (-22 – -5) | 0.021 |
